# Evidence of Transcriptional Shutoff by Pathogenic Viral Haemorrhagic Septicaemia Virus in Rainbow Trout

**DOI:** 10.3390/v13061129

**Published:** 2021-06-11

**Authors:** Irene Cano, Eduarda M. Santos, Karen Moore, Audrey Farbos, Ronny van Aerle

**Affiliations:** 1 International Centre of Excellence for Aquatic Animal Health, Cefas Weymouth Laboratory, Barrack Road, The Nothe, Weymouth DT4 8UB, Dorset, UK; ronny.vanaerle@cefas.co.uk; 2 Biosciences, College of Life and Environmental Sciences, University of Exeter, Stocker Road, Exeter EX4 4QD, Devon, UK; e.santos@exeter.ac.uk; 3 Sustainable Aquaculture Futures, Biosciences, College of Life and Environmental Sciences, University of Exeter, Stocker Road, Exeter EX4 4QD, Devon, UK; 4 Exeter Sequencing Service, Geoffrey Pope Building, University of Exeter, Exeter EX4 4QD, Devon, UK; K.A.Moore@exeter.ac.uk (K.M.); A.Farbos@exeter.ac.uk (A.F.)

**Keywords:** VHSV, transcriptomics, pathogenicity, transcriptional shutoff, rainbow trout

## Abstract

The basis of pathogenicity of viral haemorrhagic septicaemia virus (VHSV) was analysed in the transcriptome of a rainbow trout cell line inoculated with pathogenic and non-pathogenic VHSV isolates. Although both VHSV isolates showed similar viral replication patterns, the number of differentially expressed genes was 42-fold higher in cells inoculated with the non-pathogenic VHSV at 3 h post inoculation (hpi). Infection with the non-pathogenic isolate resulted in Gene Ontologies (GO) enrichment of terms such as immune response, cytokine-mediated signalling pathway, regulation of translational initiation, unfolded protein binding, and protein folding, and induced an over-representation of the p53, PPAR, and TGF-β signalling pathways. Inoculation with the pathogenic isolate resulted in the GO enrichment of terms related to lipid metabolism and the salmonella infection KEGG pathway involved in the rearrangement of the cytoskeleton. Antiviral response was evident at 12hpi in cells infected with the pathogenic isolate. Overall, the data showed a delay in the response of genes involved in immune responses and viral sensing in cells inoculated with the pathogenic isolate and suggest transcriptional shutoff and immune avoidance as a critical mechanism of pathogenicity in VHSV. These pathways offer opportunities to further understand and manage VHSV pathogenicity in rainbow trout.

## 1. Introduction

Viral haemorrhagic septicaemia (VHS) is a notifiable fish disease by the World Organization for Animal Health (OIE). From when it was first reported as a disease affecting freshwater rainbow trout in Europe in the 1930s [1], it was estimated to result in economic losses of between USD 60 and 150 million annually for the Western European and Norwegian aquaculture industry, before sanitation programmes were successfully implemented in the latest 1960s [2]. The causative agent, viral haemorrhagic septicaemia virus (VHSV) triggers a lethal infection in a range of farmed fish species, including rainbow trout *Oncorhynchus mykiss* L., turbot *Scophthalmus maximus* L., and Japanese flounder *Paralichthys olivaceus*, and has also been isolated in a broad range of asymptomatic wild fish [3,4].

VHSV is a single-stranded RNA novirhabdovirus within the family *Rhabdoviridae*. It consists of an enveloped, non-segmented, negative-sense, genome of approximately 11,200 nucleotides and encodes for five structural proteins and one non-structural protein (Nv) in the following order: 3’-nucleoprotein (N)—phosphoprotein (P)—matrix protein (M)—glycoprotein (G)—non-virion (Nv)—polymerase (L)-5’ [5]. Phylogenetic analysis grouped VHSV into four genogroups (I–IV) with genogroup I further delineated into five distinct phylogenetic subtypes (Ia to Ie), and including viruses originating in freshwater farm sites and also in marine and brackish waters [6,7]. The majority of VHSV isolates causing outbreaks in freshwater rainbow trout farms belong to sub-lineage Ia and Ic [8,9]. 

The sublineage of VHSV that is pathogenic to rainbow trout most likely emerged from a genotype I-type marine ancestor by the occurrence of a single introduction or mutation event followed by the expansion of this new genotype within trout aquaculture [10,11]. Pathogen host shifts are usually associated with changes in pathogen virulence. The viral adaptation to a new host can include nucleotide changes in the viral genome to result in adaptation to new surface receptors, new mechanisms of immune-evasion, and increased pathogen fitness [12,13]. Despite the economic impact of VHSV on the salmonid industry, very little is known about host innate immune responses to VHSV. Members of virulent VHSV genogroups Ia and Ic replicate faster in a rainbow trout gonadal cell line (RTG-2) than non-virulent Ib and II isolates [14]. A positive correlation of virulence with viral fitness has also been suggested for other novirhabdoviruses [15,16]. Viral mechanisms of immune evasion, through down-regulation of host innate and adaptive responses, have been identified in mammals and other animals [17], in particular, mammalian rhabdoviruses are thought to directly interfere with the type I interferon (IFN) system and other immune effector functions to evade immune control [18,19]. Recent studies demonstrated that virulent rainbow trout VHSV isolates inhibited gene expression of the interferon-induced GTP-binding protein Mx in vitro, suggesting that virulent isolates may interfere in the IFN pathway, potentially resulting in higher pathogenicity [14]. Further, it has been reported that the VHSV Nv protein negatively regulated host antiviral response by reducing the transcription factor NF-kappa-B activator (tank)-binding kinase 1 (tbki) phosphorylation, and therefore preventing the activation of interferon regulatory factor 3 (irf3) and the induction of type I IFN response following viral infection [20].

The present study aimed to investigate the mechanisms associated with VHSV pathogenicity in rainbow trout. We hypothesised that the molecular responses to pathogenic and non-pathogenic VHSV isolates would differ in particular during the early stages of infection. A comparison of these different molecular responses would allow for the identification of potential molecular mechanisms responsible for virulence of VHSV. To test these hypotheses, we inoculated RTG-2 cells with pathogenic and non-pathogenic isolates of VHSV and compared the transcriptomic responses over time. Our results revealed the presence of immune evasion mechanisms, and the role of the unfolded protein response (UPR) in VHSV pathogenicity, highlighting the potential for these to be used as molecular targets for novel therapeutics.

## 2. Materials and Methods

### 2.1. VHSV Cell Culture and Sampling Regime

Low passages (p4–p7) of a freshwater VHSV isolate obtained from a VHS outbreak in farmed rainbow trout, belonging to genogroup Ia (J167 [9]); and a marine VHSV, isolated from sub-clinically infected herring *Clupea harengus* belonging to the genogroup II (1p49 [21]), which was shown to be non-pathogenic to rainbow trout [22], were titrated and inoculated into RTG-2 cells (CCL55, ATCC, LGS Standards), a rainbow trout gonad derived cell line [23]. The viral multiplicity of infection (MOI) was calculated as the number of infectious units in a plaque assay (number of plaques forming units per cells (PFU/cells)) [24].

For the viral infections, approximately 10^4^ RTG-2 cells per well were plated in 12-well plates containing maintenance medium ((MM) L-15 supplemented with 1 mM L-glutamine, 10% foetal bovine serum, Gibco (Paisley, UK) and 1% penicillin-streptomycin, Merck Life Science (Gillingham, UK)). Cell monolayers were inoculated with J167 and 1p49 at 1 MOI and incubated at 15 °C. Inoculated cells were then harvested in triplicate at 3, 6 and 12 h post inoculation (pi). Negative controls consisting of non-inoculated cells were taken at 3 h pi. For each sampling point, cells were washed with phosphate-buffered saline (PBS) (Sigma, Gillingham, UK) and detached by addition of 300 µL of RLT lysis buffer (Qiagen, Manchester, UK) for subsequent RNA extraction. 

### 2.2. Library Prep and RNA Sequencing

Total RNA was extracted from each sample using an EZ1 RNA Cell Mini Kit v2.0 and EZ1 extraction robot (Qiagen, Manchester, UK). Following manufacturer’s instructions, RNase-DNase I (Qiagen Manchester, UK) was added to remove any DNA contamination during the extraction procedures. RNA was quantified using a Quantus™ fluorometer (Promega, Southampton, UK). Illumina indexed sequencing libraries were prepared from 100 ng high-quality RNA using poly-A isolation as part of the TruSeq Stranded mRNA Sample Preparation protocol (Illumina, Great Abington, UK). Tapestation analysis (Agilent, Worthing, UK) showed a library smear ranging in size from 200 bp to 900 bp with a peak at 415 bp without primer dimers. Samples were pooled in equimolar concentrations before denaturing, diluting, and 125 paired-end (PE) sequencing on an Illumina HiSeq2500 over two lanes of a high-output flowcell using v4 SBS (Sequencing by Synthesis) reagents (Illumina, Great Abington, UK). 

### 2.3. Sequence Preprocessing

The raw Illumina paired-end sequence reads generated were quality-checked using FastQC v0.11.30 [25] and subsequently trimmed to remove adapter sequences and low-quality bases using Trim Galore! v0.4.0 (www.bioinformatics.babraham.ac.uk/projects/trim_galore/). The number of reads obtained before and after quality-trimming were calculated and ANOVA tests were used to identify potential differences in read numbers between the treatments and any of the time points (*p* < 0.05).

### 2.4. De Novo Genome Assembly of the VHSV J167 Isolate

Quality-trimmed reads of one replicate sample of VHSV J167 (J167_12_3) were assembled de novo with rnaSPAdes v3.13.2 [26] (using default parameters) to obtain the full genome sequence of this isolate.

### 2.5. Reference Transcriptome

A reference rainbow trout transcriptome (NCBI Oncorhynchus mykiss Annotation Release 100) was downloaded from NCBI (https://www.ncbi.nlm.nih.gov/genome/annotation_euk/Oncorhynchus_mykiss/100; accessed on 19 October 2019) and the genome sequences of the *de novo* assembled pathogenic J167 VHSV isolate and non-pathogenic isolate 1p49 (GenBank Accession KM244767.1) were added to generate a combined rainbow trout-VHSV genome reference transcriptome. The rainbow trout transcriptome was annotated using a combination of DIAMOND v0.9.34 [27] (blastx algorithm and the ‘sensitive’ parameter; e-value < 1 × 10^−10^) and the RefSeq Release 84 database and Blast2GO 5 PRO [28]. The latter was used to identify conserved protein signatures (including trans-membrane regions, enzymes, signal peptides and structural domains) using InterProScan (see Blast2GO manual for details). These annotations were used to assign Gene Ontology (GO) terms to each of the transcripts using database version 2018.02 in Blast2GO 5 PRO. 

### 2.6. Differential Gene Expression Analysis

Transcriptome analyses were carried out using a collection of scripts comprising the Trinity RNA-Seq pipeline [29]. For each sample, transcript abundance was estimated with the align_and_estimate_abundance.pl script provided by Trinity, using RSEM v1.2.25 [30], bowtie v1.2.2 [31], the reference transcriptome and all quality-trimmed paired reads. Differences in transcript expression in cells infected with either the non-pathogenic (1p49) or the pathogenic VHSV isolate (J167) at each time point (3, 6, 12 h pi) versus the controls were determined using edgeR [32] in R Bioconductor [33]. Transcripts were considered significantly differentially expressed when the adjusted *p* value was < 0.05 (Benjamini–Hochberg method [34]) and fold-change ≥ 2. Lists of genes that were differentially expressed in cells infected with non-pathogenic (1p49) and pathogenic VHSV (J167) at 3, 6 and 12 h pi were compared and visualised as force-directed graphs using DiVenn v1.0 [35]. An html file to dynamically view lists and Venn diagrams of the differentially expressed genes produced in this study was generated using Vennt v0.8.4 (drpowell.github.io/vennt/ [36]; the link to this html file is (doi.org/10.6084/m9.figshare.14237489) and can be viewed in a web browser.

The numbers of TMM (trimmed mean of M values) normalised reads [37] that mapped to each of the VHSV genome sequences over time were plotted using the ggplot2 v.3.2.1 [38] package in RStudio v1.2.1335 to visualise temporal viral replication. A CGGC (Compare Groups of Growth Curves) permutation test was carried out (10,000 permutations) to assess differences between the curves of the pathogenic and non-pathogenic VHSV isolates [39].

Hierarchical clustering was performed and a heatmap generated by clustering the differentially expressed transcripts according to their patterns of expression across the samples (Euclidean distance similarity metric and complete linkage method) using the analyze_diff_expr.pl script (provided with Trinity). The differentially expressed transcripts were partitioned into gene clusters (sub-clusters) with similar expression patterns using the define_clusters_by_cutting_tree.pl script (based on 65 percent of the maximum height of the tree).

### 2.7. Functional Annotation Analysis

Functional annotation analyses, including Gene Set Enrichment Analysis (GSEA) based on Gene Ontology (GO) and KEGG Pathway over-representation analysis were conducted using the clusterProfiler v3.10.1 package [40] in RStudio v1.2.1335 [41]. For each treatment and timepoint combination, files were generated containing sorted rank scores (high to low values) for all transcripts in the reference transcriptome, which were calculated by multiplying the direction (sign) of fold change and the logarithm of the *p*-value for each gene. The ranked gene lists were analysed with the GSEA function within clusterProfiler using 1000 permutations, minimum and maximum gene set sizes of 10 and 500, respectively and lists containing ‘GO term to transcript ID’ and ‘GO term to GO description’ (generated by Blast2GO Pro). GO terms were considered enriched when adjusted *p* values < 0.25 (Benjamini–Hochberg method) [34]. Enriched GO terms were categorised and mapped to ancestor immune class GO terms using CateGOrizer [42] and visualised as a dotplot using ggplot2 v.3.2.1 [38] in RStudio v1.2.1335.

Kyoto Encyclopedia of Genes and Genomes (KEGG) pathway analysis was conducted in RStudio v1.2.1335 [41] using the enrichKEGG function part of clusterProfiler v3.10.1. Since there were no pathway entries for rainbow trout in the KEGG database, zebrafish (*Danio rerio*) gene orthologs and their corresponding KEGG pathway information were used instead. Orthologous zebrafish genes were obtained by blasting the rainbow trout transcripts against all zebrafish protein sequences (Ensembl Zebrafish GRCz11; Genbank Accession GCA_000002035.4) using DIAMOND v0.9.34 (blastx algorithm; e-value < 1 × 10^−10^) [27]. For each experimental condition, a list of differentially expressed genes (*p* < 0.05 and fold-change ≥ 2) containing zebrafish NCBI GeneIDs, log2 fold-change and *p* values was created and used for KEGG pathway over-representation analysis using enrichKEGG (Benjamini–Hochberg correction, FDR < 0.1) and the KEGG terms of all zebrafish orthologs in the dataset as background (universe). Gene-concept networks, displaying genes associated with significant KEGG pathways, were generated using the cnetplot function of clusterProfiler; the zebrafish gene symbols used for these plots were retrieved from the Bioconductor annotation data package org.Dr.eg.db v3.7.0 using the setReadable function of the DOSE v3.8.2 package [43].

Graphs and images were combined into single composition figures using Inkscape v0.91 (https://inkscape.org). 

### 2.8. Real-Time RT-PCR Analysis

Gene expression analysis was performed by Taqman qPCR assays for a selection of five rainbow trout genes (Table S2 in the Supplemental Material). Beta-actin was used as internal control gene to normalize the expression of the targeted genes. Briefly, 100 ng of the same RNA samples used for the RNA-Seq analysis were reverse-transcribed in a 20 µL reaction containing 1 mM dNTP, 500 ng of random hexamers and 200 units M-MLV reverse transcriptase (Promega, Southampton, GB) at 37 °C for 1 h. Taqman qPCRs were performed in duplicate with 4 µL of cDNA (containing 10 ng of input RNA), 500 nM of each primer and 250 nM of probe labelled with 6-FAM in 5’ and MGB in 3’, in a total volume of 20 µL, using the Taqman Universal PCR master Mix with AmpErase UNG (Applied Biosystem, Life Technologies, Paisley, GB). Forty cycles of qPCR and fluorescence detection were performed on a StepOne Real-Time PCR, with software V2.3 (Applied Biosystem, Life Technologies, Paisley, GB) as recommended by the manufacturer. Molecular-grade water was used as a negative control for each PCR assay. Serial ten-fold dilutions of a cDNA sample were used to determine the efficiency of each set of primers, giving slope values close to −3.4. Normalized relative quantities (CNRQ) in gene expression, equivalent to the fold change method (2∆∆Ct), were calculated using qbase + software, version 3.2 [44]. 

## 3. Results

### 3.1. Transcriptome Sequencing Mapping and Annotation

In total, 189,153,793 paired sequence reads were obtained and the number of paired reads for the various samples ranged from 7.1 to 10.9 million (7.0 to 10.7 million after quality filtering; Table S1 in the Supplemental Material). There were no significant differences between the numbers of reads for any of the treatment groups, both before and after quality-trimming (*p* = 0.41 and 0.43, respectively). 

A publicly available transcriptome (NCBI Oncorhynchus mykiss Annotation Release 100) was retrieved and re-annotated to allow for subsequent Gene Set Enrichment Analysis based on Gene Ontology (GO) and interpretation of differentially expressed transcripts. A total of 76,438 transcripts (96.5% of the transcriptome) were annotated against the RefSeq protein database (e-value < 1 × 10^−10^) and GO terms were assigned to 61,073 of these transcripts (Dataset S1 in the Supplemental Material). 

### 3.2. At Early Times Post Inoculation, Pathogenic and Non-Pathogenic VHSV Isolates Replicate in a Similar Manner in RTG-2 Trout Cells Despite Large Differences in the Host Responses 

**Figure 1:**
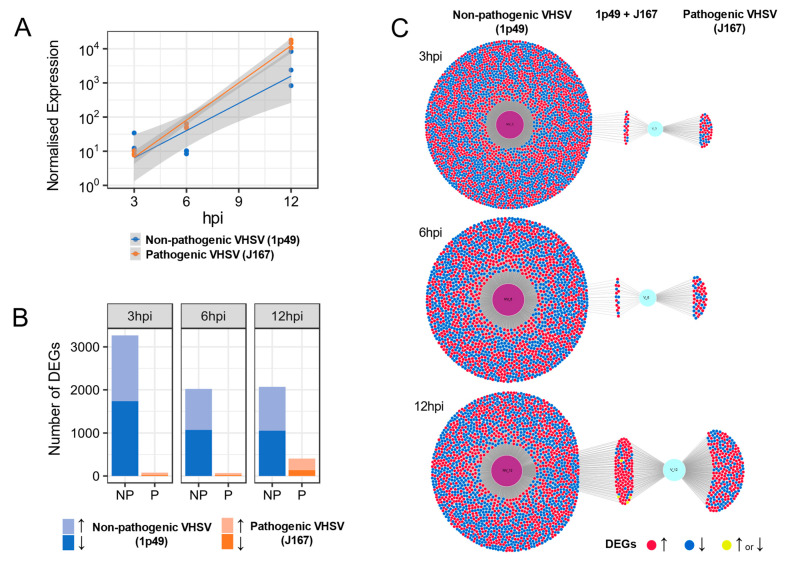
Gene expression of the rainbow trout gonad cell line RTG-2 inoculated either with a non-pathogenic (1p49; NP) or a pathogenic (J167; P) viral haemorrhagic septicaemia virus (VHSV) isolate. (**A**). Normalised viral sequence reads at 3, 6, and 12 h post-inoculation (hpi). (**B**) Number of differentially expressed genes (DEGs) in RTG-2 cells over time inoculated either with the non-pathogenic or pathogenic isolates. Arrows indicate whether the genes were up-regulated or down-regulated. (**C**) Graphical overview of the number of differentially expressed host transcripts in cells inoculated with each viral isolate and a representation of overlapping host transcripts differentially expressed following inoculation with each isolate.

We mapped the sequence reads to the VHSV genome to assess the level of replication of each VHSV isolate and the number of normalised reads mapping to the VHSV genome over time is shown in Figure 1A. Both VHSV isolates showed similar patterns, with an exponential increase in the number of normalised sequenced reads from 3 to 12 h pi and no significant differences were observed between the curves (adjusted *p* = 0.099) (Figure 1A). In contrast, the number of differentially expressed host transcripts in cells inoculated with the non-pathogenic isolate (1p49) was far greater than that for cells inoculated with the pathogenic isolate (J167; Figure 1B,C; Dataset S2 in the Supplemental Material). The greatest difference was seen at 3 h pi, where the numbers of differentially expressed genes (DEGs) were 3266 for the non-pathogenic and 77 for the pathogenic isolate, a difference of approx. 42-fold (Figure 1B,C). There was little overlap between differentially expressed genes in the cells inoculated with each VHSV isolate over time. At 12hpi, overlapping genes were over-expressed in both the pathogenic and non-pathogenic treatment groups (Figure 1C).

### 3.3. Hierarchical Clustering Reveals That Pathogen Virulence Is the Main Determinant of Differential Transcription Profiles

**Figure 2:**
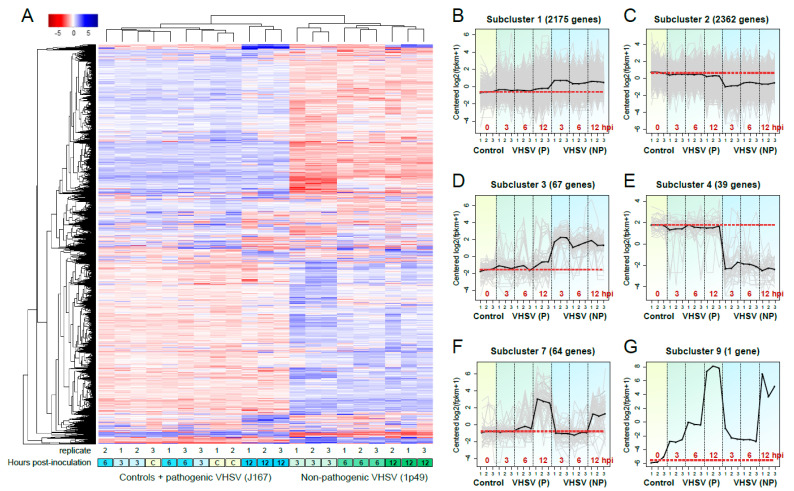
(**A**) Heatmap showing clustering of all differentially expressed genes in the rainbow trout gonad cell line RTG-2 inoculated with either the non-pathogenic (NP) 1p49 or the pathogenic (P) J167 viral haemorrhagic septicaemia virus (VHSV) isolates at 3, 6, and 12 h post-inoculation. (**B**–**G**) Partitioning of differentially expressed genes into gene clusters with similar expression profiles over time.

Clustering analysis based on the list of all differentially expressed genes revealed that all samples inoculated with non-pathogenic VHSV (1p49) clustered together and clearly separated from samples inoculated with the pathogenic VHSV (J167), which clustered together with the controls for the 3 and 6h time points (Figure 2A). Further, for the non-pathogenic VHSV, samples clustered according to the time points at which they were collected, revealing a strong influence of time on the expression patterns observed (Figure 2A). In contrast, for samples inoculated with the pathogenic isolate, there was little separation between samples belonging to each time point, with the exception of samples collected after 12hpi, which clustered together (Figure 2A).

Partitioning of differentially expressed genes into gene clusters with similar expression profiles resulted in 9 sub-clusters (Figure 2B–G; Gene clusters in Dataset S2 in the Supplemental Material). Of these, 6 clusters showed clear differences in expression patterns between the 2 VHSV isolates, with sub-clusters 1 and 3 (Figure 2B,D) showing up-regulation of genes for the non-pathogenic VHSV including the antimicrobial peptides (AMPs) *cathelicidin antimicrobial peptide* (camp) (fold change (FC) = 6.8; false discovery rate (FDR): 6.7 × 10^−25^) and the acute-phase protein *serum amyloid A-5 protein*-like (*saa5*) (FC = 4.4; FDR: 1.6 × 10^−17^), and genes related with metabolic processes, such as *palmitoleoyl-protein carboxylesterase*-like (*notum*) (FC = 2.1; FDR: 1.8 × 10^−7^). In contrast, sub-clusters 2 and 4 (Figure 2C,E) showed down-regulation of genes for the non-pathogenic VHSV related with lipid metabolism, including *low density lipoprotein* (*ldp*) (FC = −7.2; FDR: 6.0 × 10^−4^), and cytoskeleton, including the *tropomyosin alpha-1 chain*-like (*tpm1*) isoform X1 (FC = −8.4; FDR: 4.3 × 10^−8^).

Sub-cluster 7 (Figure 2F) represented a selection of genes (64 in total) that were up-regulated at 12hpi for both VHSV subtypes. This sub-cluster included genes related with biotic stimuli and immune responses as the IFN-inducible *mx* gene isoform 2 (*mx2*) (FC = 3.4 and 4.0; FDR: 9.5 × 10^−10^ and 5.0 × 10^−15^ for 1p49 and J167, respectively) and *mx* gene isoform 3 (*mx3*) (FC = 4.8 and 5.8; FDR: 2.5 × 10^−4^ and 6.7 × 10^−8^ for 1p49 and J167, respectively), and specific fish virus induced tripartite motif (TRIM) proteins such as *VHSV induced genes* 3, 4 and 5 (*vig-1*, *vig-4*, and *vig-5*) (for *vig-4*: FC = 7.1 and 8.2; FDR: 7.2 × 10^−22^ and 3.2 × 10^−33^ for 1p49 and J167, respectively)]. Genes related to the extracellular region, such as the extracellular matrix glycoprotein *fibronectin 1* (*fn1*) (FC = 11.8 and 11.8; FDR: 1.9 × 10^−5^ and 6.1 × 10^−45^ for 1p49 and J167, respectively) were also up-regulated. Sub-cluster 9 (Figure 2G) showed the expression of VHSV sequences, which increased several orders of magnitude over time. 

### 3.4. Gene Set Enrichment Analyses (GSEA) and KEGG Pathway Enrichment Reveal Delayed Activation of Immune Response Pathways in Cells Inoculated with the Pathogenic VHSV Isolate

**Figure 3:**
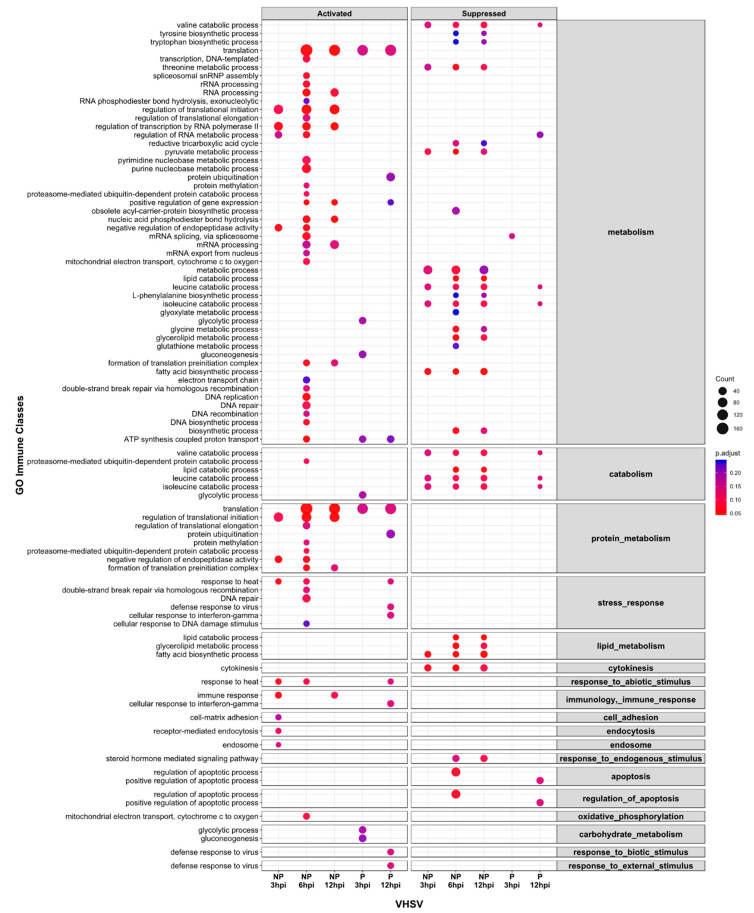
Dot plot of grouped enriched gene ontology (GO) terms into immune classes for the non-pathogenic (NP) 1p49 and pathogenic (P) J167 viral haemorrhagic septicaemia virus (VHSV) isolates at 3, 6, and 12 h post-inoculation (hpi), based on gene set enrichment analysis of all transcripts. The size and colour of each dot represent the number of genes and adjusted *p* values (Benjamini–Hochberg correction; FDR < 0.25), respectively.

Gene Set Enrichment Analysis identified a total of 169 unique GO terms enriched over all conditions (see Dataset S3 in the Supplemental Material) and 62 of these belonged to at least one of the 18 ancestor immune class GO terms (Figure 3; Dataset S4 in the Supplemental Material). Interestingly, enriched GO terms were identified for all time points in the lists of differentially expressed genes for cells inoculated with the non-pathogenic 1p49 but in contrast, for cells inoculated with the pathogenic J167, enriched GO terms were only present at 3 and 12hpi (Figure 3; Datasets S3,4 in the Supplemental Material).

A key feature of the list of enriched GO terms was the evidence of response to the pathogen as early as 3hpi in cells inoculated with the non-pathogenic 1p49 isolate, including immune response (GO:0006955; FDR = 0.05), cytokine-mediated signalling pathway (GO:0019221; FDR = 0.10), endosome (GO:0005768; FDR = 0.14), regulation of translational initiation (GO:0006446. FDR = 0.09), unfolded protein binding (GO:0051082, FDR = 0.05), and protein folding (GO:0006457, FDR = 0.05). In contrast, these processes were not enriched in cells inoculated with the pathogenic J167, and evidence of an initial immune response was only evident at 12hpi. Examples of genes that were differentially regulated in cells inoculated with the non-pathogenic 1p49 at 3hpi, and indicative of an early immune response to the virus include immunomodulators such as *proteoglycan 4* (*prg4*)-like (FC = 5.53; FDR = 6.4 × 10^−18^); chemokine and cytokine activity including *leukaemia inhibitory factor receptor* (*lifr*) (FC = 3.6; FDR = 3.1 × 10^−16^); genes involved in the interferon-stimulated JAK-STAT signalling pathway such as *suppressor of cytokine signalling 3* (*socs3*) (FC = 3.3; FDR = 7.6 × 10^−17^); genes involved in antigen presentation and inflammatory responses, as the *beta-2-microglobulin*-like (*b2m*) (FC = 7.3; FDR = 9.8 × 10^−4^), the transmembrane *C-type lectin domain family 4 member M* (*cd209*) (FC = 3.2; FDR = 1.5 × 10^−16^), *fos-related antigen 1*-like (*fosl1*) (FC = 2.9; FDR = 2.6 × 10^−11^), and *perforin 1* (*prf1*) (FC = 2.6; FDR = 3.8 × 10^−9^); genes related with transcriptional processes and endosomal sorting as *E3 ubiquitin-protein ligase ZNRF1* (*znrf1*) (FC = 3.0; FDR = 3.3 × 10^−12^), *mRNA decay activator protein ZFP36*-like (*zfp36*) (FC = 2.9; FDR = 3.2 × 10^−13^); translation initiation factors such as *eukaryotic translation initiation factor 4 gamma 2* isoform X1 (*eif4g2*) (FC = 2.5; FDR = 2.0 × 10^−10^); and genes involved in the unfolded protein response (UPR) as the *CCAAT/enhancer-binding protein β* (*c/ebpβ*) (FC = 2.3; FDR = 8.2 × 10^−9^ at 3hpi), the *negative regulator 78 kda glucose regulated protein* or *binding immunoglobulin protein* (*grp78/bip*) (FC = −2.2; FDR = 3.4 × 10^−6^ at 3hpi), and *cyclic AMP-dependent transcription factor ATF-6 alpha*-like (*atf6*) (FC = −1.4 and −2.5; FDR = 2.7 × 10^−2^ and 4.8 × 10^−2^ at 6 and 12hpi, respectively). None of these genes were differentially expressed in cells inoculated with the pathogenic J167 before 12hpi, revealing a delay in the regulation of immune genes and viral sensing for the cells inoculated with the pathogenic isolate. 

The GO term response to heat (GO:0009408), as a response to external stimulus or cellular stress, was activated in cells inoculated with the non-pathogenic VHVS at all sampling points (FDR = 0.05, 0.09, 0.15 at 3, 6, and 12hpi, respectively), but only at 12hpi in cells inoculated with the pathogenic isolate (FDR = 0.14). Examples of heat shock proteins (HSPs) differentially transcribed among isolates included *heat shock protein 90 alpha* (*hsp90aa1*) (FC = 4.1; FDR = 2.6 × 10^−24^) and *heat shock protein 30* (*hsp30*) (FC = 7.2; FDR = 6.6 × 10^−26^), which were up-regulated by the non-pathogenic 1p49 as early as 3hpi. However, HSPs of family 70 were greatly induced by the pathogenic VHSV at 12hpi, including *heat shock protein 70b* (*hsp70b*) (FC = 7.4; FDR = 1.2 × 10^−52^), *heat shock protein 70a* (*hsp70a*) (FC = 6.2; FDR = 9.1 × 10^−33^), *heat shock 70 kDa protein*-like (*hsp70*) (FC = 6.7; FDR = 1.7 × 10^−11^), and *DnaJ homolog subfamily B member 1* (*dnajb1*) (FC = 5.4; FDR = 7.0 × 10^−25^).

Supressed GO terms for the non-pathogenic virus were related with the regulation of cytoskeleton including the GO terms tubulin complex (GO:0045298, FDR = 0.05, 015, and 0.04 at 3, 6, and 12hpi, respectively) and microtubule (GO:0005874, FDR = 0.05, 0.2, and 0.17 at 3, 6, and 12hpi, respectively). In addition, inoculation with the non-pathogenic isolate also suppressed other processes including metabolic processes (GO:0008152, FDR = 0.13, 0.07, and 0.2 at 3, 6, and 12hpi, respectively), transmembrane transport (GO:0055085, FDR = 0.04 and 0.09 at 6, and 12hpi, respectively), gycerolipid metabolic process (GO:0046486, FDR = 0.05, and 0.08 at 6 and 12hpi, respectively) and lipid catabolic process (GO:0016042, FDR = 0.05, 0.04, and 0.04 at 3, 6 and 12hpi, respectively). Within these GO terms, example of genes down-regulated in cells inoculated with the non-pathogenic 1p49 at 3hpi included *ras-related protein Rab-3D* (*rab3d*) (FC = −2.1; FDR = 5.2 × 10^−5^), *tubulin alpha chain* (*tuba1a*) (FC = −3.9; FDR = 1.2 × 10^−20^), *sterol regulatory element-binding protein-2* (*srebf2*) (FC = −2.1; FDR = 7.8 × 10^−4^), *low-density lipoprotein receptor* (*ldlr*) (FC = −5.7; FDR = 2.2 × 10^−7^), the *tetraspanin CD9 antigen*-like (*cd9*) (FC = −4.4; FDR = = 1.5 × 10^−11^), and the *OX-2 membrane glycoprotein-like isoform X4* (*cd200*) (FC = −5.6; FDR = 3.1 × 10^−25^).

Interestingly, while the non-pathogenic VHSV suppressed double-stranded RNA binding pathway (GO:0003725, FDR = 0.1 and 0.05 at 3 and 6hpi, respectively) and down-regulated *cAMP-dependent protein kinase inhibitor gamma* (*pkig*)-like isoform X1 at 6 and 12h (FC = −1.3 and −1.2; FDR = 8.6 × 10^−3^ and 4.4 × 10^−2^), the pathogenic isolate induced its up-regulation at 12hpi (FC = 9.3; FDR = 5.4 × 10^−14^) together with up-regulation of *double-stranded RNA adenosine deaminase activity* (*adar*) (FC = 2.5; FDR = 2.4 × 10^−4^), interferon-induced double-stranded RNA-activated *protein kinase* (*pkr*) or *eukaryotic translation initiation factor 2 alpha kinase 2* (*eif2ak2*) (FC = 3.4; FDR = 1.05 × 10^−6^), and *BAG family molecular chaperone regulator 3* (*bag3*) (FC = 4.1; FDR = 1.4 × 10^−7^).

**Figure 4:**
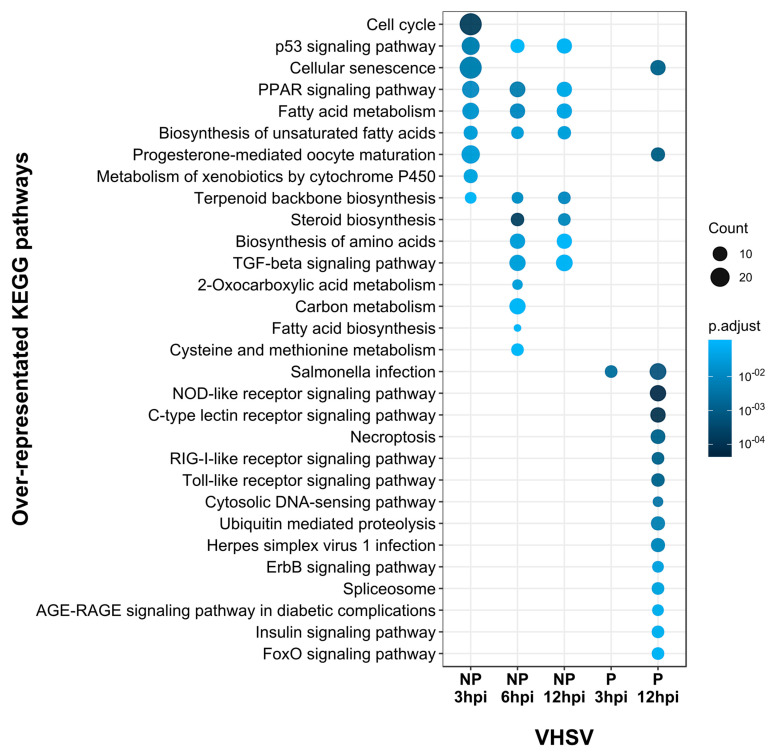
Dot plot of enriched Kyoto Encyclopedia of Genes and Genomes (KEGG) pathways for the non-pathogenic (NP) 1p49 and pathogenic (P) J167 viral haemorrhagic septicaemia virus (VHSV) isolates at 3, 6, and 12 h post-inoculation (hpi), based on lists of differentially expressed genes (*p* < 0.05 and fold-change ≥ 2) using zebrafish orthologs of rainbow trout transcripts. KEGG pathway over-representation analysis was performed using enrichKEGG (Benjamini–Hochberg correction, FDR < 0.1) and the KEGG terms of all zebrafish orthologs in the dataset as background (universe). The size and colour of each dot represent the number of genes and adjusted *p* value (Benjamini–Hochberg correction; FDR < 0.1), respectively.

**Figure 5:**
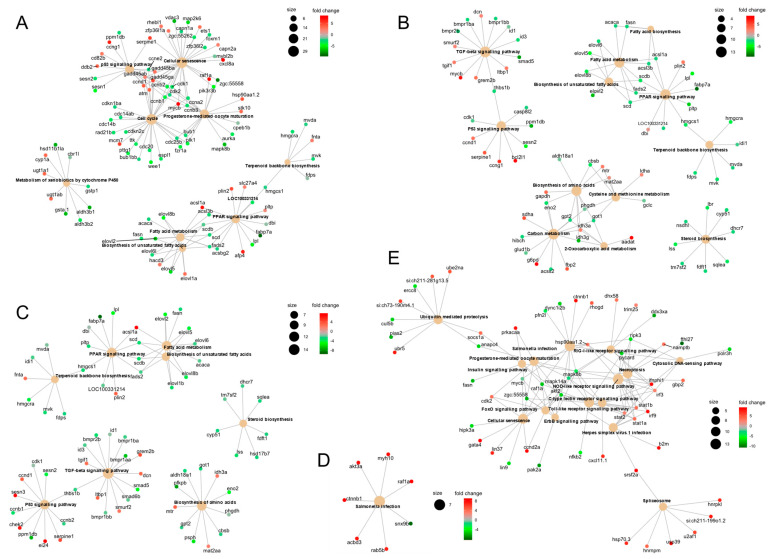
(**A**–**E**) Gene-concept networks showing genes associated with enriched KEGG pathways in the rainbow trout gonad cell line RTG-2 inoculated with non-pathogenic viral haemorrhagic septicaemia virus (VHSV) (1p49) at 3 h post-inoculation (hpi) (**A**), 6hpi (**B**), and 12hpi (**C**); and pathogenic VHSV (J167) at 3hpi (**D**) and 12hpi (**E**). The sizes of the category nodes (enriched KEGG pathways) are proportional to the number of genes and the colours of the gene nodes represent log2 fold-change (red = up-regulated genes, green = down-regulated genes).

Analysis of over-representation of KEGG pathways, similarly to that for GSEA, identified over-represented pathways among the differentially expressed gene lists for cells inoculated with the non-pathogenic 1p49 at all time points analysed (Figure 4 and Figure 5; Dataset S5 in the Supplemental Material). In contrast, for cells inoculated with the pathogenic J167, over-represented pathways were only identified among the differentially expressed genes 12h after inoculation, with the exception of salmonella infection KEGG pathway identified also at 3hpi (*p* = 0.001, *p* = 0.04 at 3 and 12hpi, respectively) (Figure 4 and Figure 5; Dataset S5 in the Supplemental Material). There was little overlap between the KEGG pathways over-represented for the pathogenic and non-pathogenic isolate, in line with the small overlap in the lists of DEGs described above. Of note, inoculation with the pathogenic J167 induced antiviral responses mediated by the activation of C-type lectin, nucleotide-binding oligomerization domain (NOD)-like, retinoic acid-inducible gene I (RIG-I)-like, and Toll-like receptor signalling pathways (*p* = 6.79 × 10^−5^, *p* = 5.19 × 10^−5^, *p* = 8.81 × 10^−4^ and *p* = 8.81 × 10^−4^, respectively; Figure 4), but these pathways were only activated late in the infection process (12hpi) and were not over-represented for the non-pathogenic isolate. As examples, genes that were differentially regulated in cells inoculated with the pathogenic J167 at 12hpi included *irf3 isoform* x1 (FC = 2.3; FDR: 1.4 × 10^−6^), *signal transducer and activator of transcription 1-alpha/beta-like* isoform X2 (*stat1b*) (FC = 3.3; FDR = 7.8 × 10^−7^), and *C-X-C motif chemokine 11* (*cxcl11*) (FC = 3.1; FDR = 1.4 × 10^−11^) (Figure 5).

Inoculation with the non-pathogenic 1p49 resulted in over-representation of the p53 signalling pathway (dre04115) at all time points (*p* = 4.0 × 10^−4^, *p* = 0.098, *p* = 0.075, respectively; Figure 4). In addition, several pathways related to metabolism were over-represented for one or more time points in cells inoculated with the non-pathogenic 1p49, but not for cells inoculated with the pathogenic J167, including, peroxisome proliferator-activated receptor (PPAR) signalling pathway (*p* = 9.6 × 10^−3^, *p* = 4.4 × 10^−3^ and *p* = 0.045 for 3, 6, and 12hpi, respectively), fatty acid metabolism (*p* = 0.015, *p* = 0.009 and *p* = 0.037 for 3, 6, and 12hpi, respectively), and transforming growth factor-β (TGF- β) signalling pathway (*p* = 0.026 and *p* = 0.074 for 6 and 12hpi, respectively). Genes that were differentially regulated in cells inoculated with the non-pathogenic 1p49 at 3hpi included the *plasminogen activator inhibitor 1*-like (*serpine1*) (FC = 5.1; FDR = 4.5 × 10^−34^) and the *growth arrest and DNA damage-inducible protein GADD45 alpha* (*gadd45a*) (FC = 2.0; FDR = 7.5 × 10^−6^) for the p53 pathway; general down-regulation of genes involved in the PPAR and fatty-acid metabolism pathways such as *acyl-CoA synthetase family member 2, mitochondrial*-like (*acsl1a*) (FC = −1.2; FDR = 1.7 × 10^−2^), *hydroxymethylglutaryl-CoA synthase, cytoplasmic*-like (*hmgcs1*) (FC = −2.0; FDR = 5.7 × 10^−7^), and *acetyl-CoA carboxylase 1* (*acaca*) (FC = −2.4; FDR = 5.0 × 10^−5^); and up-regulation of genes involved in the TGF- β pathway as the *latent-transforming growth factor beta-binding protein 2*-like (*ltbp2*) (FC = 3.1; FDR = 6.5 × 10^−13^) and *decorin* (*dcn*) (FC = 3.6; FDR = 3.8 × 10^−10^) (Figure 5).

### 3.5. Quantitative PCR Validation of Differential Expression Data

Relative quantification of the transcription profiles for five genes identified to be differentially expressed in the RNA-Seq dataset between the isolates is presented in Figure S1 in the Supplemental Material. The gene expression patterns obtained with the qPCRs in general followed the same trend observed in the RNA-Seq analysis, validating the accuracy of the differential expression analysis in the RNA-Seq dataset.

## 4. Discussion

### 4.1. Transcriptomics Showed Evidence of Cellular Transcriptional Shutoff by the Pathogenic VHSV

We observed that both VHSV isolates showed a similar pattern of viral replication at early times post-inoculation, but the induced host cell response to each viral isolate was dramatically different as shown at 3hpi, where the number of DEGs were 42-fold higher in cells inoculated with the non-pathogenic VHSV. GO terms related to transcription, translation, and mRNA processes were activated only in cells inoculated with the non-pathogenic isolate, suggesting that the pathogenic VHSV impairs transcription to prevent the production of antiviral proteins and host response, inhibiting, therefore, transcription-dependent host cell defences, a process known as shutoff [45]. Similarly, it has been shown that other rhabdoviruses, as the vesicular stomatitis virus (VSV), inhibit in vitro host protein synthesis between 3 and 6hpi by inhibition of host translation initiation factors, in particular by dephosphorylation of the eukaryotic translation initiation factor 4E (eIF4E) [46]. Indeed, a reverse genetic system for VHSV using recombinant plasmids [47] has shown a role of viral genes in promoting host shutoff. Analysis on transfected cells showed that the M protein of VHSV genogroup IVb and the fish novirhabdovirus infectious hematopoietic necrosis virus (IHNV) can mediate down-regulation of host transcription [48,49] and that the VHSV IVb non-structural Nv protein promotes phosphorylation of eukaryotic translation initiation factor 2a (eif2a) via the protein kinase RNA-like endoplasmic reticulum kinase (perk)-eIF2a pathway to regulate host shutoff and host cell response [50]. Our data further suggests that the pathogenic VHSV promotes phosphorylation of eif2a which in turn inhibits protein synthesis as evidenced by the gene up-regulation of *eif2ak2* (which is activated after binding to dsRNA promoting phosphorylation of eif2a), *adar* (which is an RNA editor induced by type I IFN involved in the regulation of translational initiation by eif2a phosphorylation [51,52]), and *pkig*-like isoform *X1* (which is a potent protein kinase inhibitor) likely increasing virus replication and inhibiting host protein synthesis. 

### 4.2. Pathogenicity May Be Associated with Delayed Viral Sensing by Pathogenic VHSV

An early classical up-regulation of innate immune responses and cytokine-mediated signalling was observed in cells inoculated with non-pathogenic VHSV as early as 3hpi, while antivirus responses were only observed at 12hpi for the pathogenic isolate. In particular, the non-pathogenic VHSV induced an earlier activation of cytokines, chemokines, immunomodulators, complement, HSPs, antigen processing and presentation by the major histocompatibility complex (MHC) class I molecules, and IFN signalling than the pathogenic VHSV isolate as seen by the consistent up-regulation of key pro-inflammatory genes including *prf1*, *fosl1*, *c7*, *prg4*, and *cd209*-like, the component of mature class I *b2m*, and potent antimicrobial peptides as *camp* [53] and *saa* [54], and down-regulation of *cd200*, involved in the suppression of inflammatory responses [55]. Limiting MHC-I cell surface expression is a strategy used by many viruses to evade immune recognition, as for influenza virus [56].

Significant differences in the regulation of HSPs among isolates were also observed: while non-pathogenic VHSV induced earlier gene expression of *hsp90* and *hsp30*, the pathogenic VHSV induced later HSPs belonging to the 70-family and their chaperones, such as *bag3* and *dnajb1*, involved in the chaperone response of the folding of unfolded proteins. HSPs are chaperones for immunogenic peptides and Toll-like receptors, provide activation signals for antigen-presenting cells and natural killer cells, interfere with uncontrolled protein unfolding, and prevent pathogen protein aggregation [57]. However, HSPs can also support and enhance viral gene expression [58,59]. Indeed, it has been shown that viruses can recruit Hsp70 chaperones to enhance viral protein folding [60] and that rabies virus infection induces the expression of Hsp70 to enhance viral replication [61]. The evidence provided here, therefore, highlights immune evasion as an important putative mechanism associated with VSHV pathogenicity and suggests that the pathogenic VHSV utilised hsp70-family and its chaperones as pro-viral factors more efficiently than the non-pathogenic isolate.

### 4.3. VHSV Pathogenicity Is Associated with Endoplasmic Reticulum Stress and Attenuation of Unfolded Protein Response (UPR)

The present study showed that unfolded protein binding and protein folding processes were activated only in cells inoculated with the non-pathogenic VHSV despite both viruses replicating at the same rate. The accumulation of large amounts of viral glycoproteins induces a series of signalling cascades causing endoplasmic reticulum stress and UPR. UPR then transduces into a programme of cellular transcriptional and translational responses ending in up-regulation of the molecular chaperone GRP78/BiP which plays a pivotal role as the master negative regulator of UPR [62]. Viruses can manipulate the UPR in order to attenuate antiviral responses [63]. Recent studies showed that Dengue virus (DENV) and enterovirus 71 (EV71) infection induces up-regulation of the GRP78/BiP which promotes viral replication [64]. Our data show a noteworthy down-regulation of *grp78/bip*, *atf6*, and *cr3b3l3* transcripts by the non-pathogenic isolate and up-regulation of *eif2ak2* by the pathogenic isolate, which strongly suggests a role for UPR in the VHSV pathogenicity. However, to fully map VHSV disruption of UPR, a longer time series (including 24 and 48hpi) is required to follow the pattern of gene transcription and translational regulation of key components of UPR, such as *IRE1*, *ATF6*, and *XBP1* [65], over time.

### 4.4. Non-Pathogenic VHSV Replication Triggered Host Stress Response Via the p53 and TGF-β Pathways

Interestingly, cells inoculated with the non-pathogenic VHSV responded to the viral-induced stress by activating p53 and TGF-β signalling pathways at early time points post-inoculation, a response that was not observed in cells inoculated with the pathogenic isolate, suggesting that the pathogenic VHSV might reduce or delay their activation. p53 and TGF-β pathways interact in cell cycle signalling, can initiate apoptosis and autophagy to block viral replication and dissemination, and are highly regulated and modulated by crosstalk with other signalling networks [66,67]. Both VHSV isolates induce cytopathic effects (CPEs) at later stages of the infection, achieving viral titres of ~10^7^ TCID_50_/mL for the pathogenic J167 and ~10^4^ TCID_50_/mL for the non-pathogenic 1p49 at seven days pi, with larger plaque sizes produced by the pathogenic one [14]. Although in the present dataset, the regulation of apoptotic processes was not seen at early times post-inoculation, the early activation of p53 and TGF-β signalling pathway in cells inoculated with the non-pathogenic VHSV potentially induced growth arrest, and this is supported by up-regulation of p53 inducible genes as *gadd45a*. This result suggests that the cell damage caused by the replication of the non-pathogenic virus likely is a result of apoptotic processes induced by p53 growth arrest to limit the spread of the infection to neighbour cells [68]; while necrosis and cell lysis induced by replication of the pathogenic one likely it is a result of host shutoff and viral release [69]. Some viruses can manipulate p53 signalling by the interference of p53 directly through viral proteins or indirectly mediated by E3 ubiquitin-protein ligase (HDM2) or induction of type I IFN to enhance viral replication [70]. Indeed, recent studies showed that knock-out of p53 promotes viral replication for certain viruses, including for SARS-CoV [71]. Our study also showed that *serpine1*, a gene regulated by TGF-β and p53 pathways, was up-regulated in cells inoculated with the non-pathogenic VHSV. It has been shown that SERPINE1 can inhibit the viral replication of some viruses, including Hepatitis C virus (HCV) [72,73]. The present data, therefore, highlight an early host activation of p53 and TGF-β signalling pathways in response to the non-pathogenic VHSV, potentially limiting further viral replication and spread. This finding requires further studies focused on the potential use p53 and some of its key target genes, including *serpine1*, as antiviral agents to suppress disease in fish rhabdoviral infections. 

### 4.5. Differential Modulation of Transcription of VHSV Receptors in Pathogenic and Non Pathogenic Isolates

We have identified alterations in the transcription of known (such as *fn1*) and novel (such as *cd9*) VHSV cellular receptors. VHSV uses the matrix glycoprotein *fn1* as a cellular receptor for cell entry [74]. Rhabdovirus adsorption is mediated by G protein attachment to cell surface receptors and penetration of the cell occurs by endocytosis via coated pits [75]. Signal transduction pathways, small G proteins, and binding activity of integrins are regulated by the tetraspanin CD9 [76] which is a necessary factor and cellular receptor for a wide number of viruses [76,77]. Our data showed that although *fn1* was up-regulated in cells inoculated with both viral isolates, *cd9* was down-regulated only in cells inoculated with the non-pathogenic, likely decreasing host susceptibility to the non-pathogenic viral isolate.4.6. Pathogenic VHSV potentially manipulated viral trafficking via alterations in host cytoskeleton dynamics. 

Activation of GO terms related to the cytoskeleton and over-representation of salmonella infection KEGG pathway was observed in cells inoculated with the pathogenic isolate early after the inoculation, while cytoskeleton terms were suppressed in cells inoculated with the non-pathogenic VHSV involving *rab3d*, *tuba1a*, and *kinesin*-like proteins. The cellular cytoskeleton provides the basis for intracellular movements of the pathogen from the cell surface to the nuclear region [78]. In particular, over-representation of salmonella infection KEGG pathway by a pathogen is characterised by a rearrangement of the actin cytoskeleton to promote pathogen uptake into host cells and its subsequent proliferation and intercellular spread, thereby enhancing virulence [79]. Similar findings were reported for the human immunodeficiency virus (HIV) which can bind directly to actin filaments and manipulate actin regulatory proteins [80]. Our data showed that infection with pathogenic VHSV modulates the cytoskeleton as a virulence mechanism which potentially facilitates viral trafficking, replication, and spread. The strategy used by VHSV to manipulate the actin cytoskeleton requires further research. 

### 4.6. Suppression of Cholesterol Biosynthesis and Trafficking as a Potential Mechanism of Attenuation of VHSV Virulence

GO terms related to the lipid metabolism were suppressed in cells inoculated with non-pathogenic VHSV. Among those down-regulated genes were *srebf2* and *ldlr*. *Srebf2* is a key transcription factor regulating the cholesterol biosynthesis pathway. Recent studies have shown that cholesterol plays an essential role in the life cycle of enveloped viruses. For example, HIV activates SREBP2 transcription [81], while HCV Ns 4B protein modulates SREBF via the AKT pathway which up-regulates the viral RNA translation [82]. LDLR proteins are involved in lipoprotein trafficking and mediate the endocytosis of cholesterol-rich LDL. Viruses, including HCV, stimulate LDLR expression to facilitate viral propagation [83]. Thus, our results showed that in cells inoculated with the non-pathogenic VHSV, cholesterol biosynthesis and trafficking processes were suppressed, suggesting that this is utilised as a mechanism to suppress viral replication in the non-pathogenic VHSV. 

### 4.7. Limitations of this Study

This study provides novel and important data to understand the virulence mechanism of VHSV in rainbow trout, but it presents some limitations. The study focused on host response to VHSV soon after the viral inoculation to reveal early changes associated with pathogenicity. This has successfully uncovered key differences in transcriptional profiles for pathogenic and non-pathogenic VHSV at the critical early stages of infection. However, analysis of later stages of infection might offer a different view with a higher presence of host anti-viral responses as seen in previous work [84,85]. Furthermore, cells were inoculated at a low MOI, which potentially results in non-infected cells in the infected group, generating variability when analysing the transcriptomic data. Single-cell sequencing approaches might offer a path to overcome this problem [86]. Finally, transcriptomic analysis can only offer a snapshot of the transcribed genes at a given time point, and not a direct indication of the level of protein expression or function [87]. Therefore, our work proposes a hypothesis for the pathways involved in pathogenicity in VHSV, which require further experimental validation for their significance and applicability to combat disease, but this was outside the scope of this study.

## 5. Conclusions

This study provides novel evidence that pathogenic VHSV utilizes immune evasion mechanisms and cellular shutoff at the early stages of infection, preventing host sensing of viral replication and the activation of P53 and TGF-β signalling pathways. Moreover, pathogenic VHSV manipulates UPR and immune responses, and modulates cytoskeleton and lipid metabolism likely to increase viral uptake, replication, and spread. Our findings provide a greater understanding of the pathogenic mechanisms of fish rhabdoviruses and can be used to evaluate potential virulence of novel (marine and freshwater) isolates and facilitate the design of future vaccine strategies antivirals.

## Data Availability

The genome sequence for J167 (UK-J167) is available on Genbank under Accession Number MW507000. The raw Illumina sequencing files were deposited in the NCBI Sequence Read Archive (SRA) under BioProject PRJNA627417.

## Supplementary Materials

The following are available online at https://www.mdpi.com/article/10.3390/v13061129/s1, File S1: The number of Illumina read sequences obtained before and after quality-trimming (Table S1); Summary of the rainbow (*Oncorhynchus mykiss*) genes and the nucleotide sequences of the primers and probes used for the Taqman qPCR assays (Table S2); Comparison of relative gene expression (fold-change) over time (3, 6 and 12 h post inoculation) of rainbow trout cells (RTG-2) inoculated either with a pathogenic (J167) or a non-pathogenic (1p49) VHSV isolate, relative to control cells, by Taqman qPCR analysis or Illumina HiSeq sequence analysis (Figure S1), File S2: Rainbow trout gene sequences annotated using DIAMOND blastx searches against the RefSeq 84 database (cutoff e-value < 1e^−10^). Conserved protein signatures, including trans-membrane regions, enzymes, signal peptides and other structural domains were predicted using InterProScan (part of the Blast2GO 5 PRO software package). Gene ontologies were assigned by Blast2GO 5 PRO (database version 2018.02) and closest matches to zebrafish orthologues were determined using DIAMOND blastx searches against the full Ensembl Zebrafish GRCz11 proteome (Dataset 1), File S3: Lists of differentially expressed genes in RTG-2 cells inoculated with non-pathogenic (1p49) and pathogenic (J167) VHSV isolates at 3, 6 and 12 h post-inoculation (adjusted *p* value < 0.05 and fold-change ≥ 2). Differentially expressed transcripts with similar expression patterns were grouped into sub-clusters using the define_clusters_by_cutting_tree.pl script (part of the Trinity RNA-Seq pipeline) and these sub-clusters are indicated in the table (Dataset 2). File S4: Enriched Gene Ontologies identified by Gene Set Enrichment Analysis (GSEA) based on ranked gene lists for non-pathogenic (1p49) and pathogenic (J167) VHSV at 3, 6 and 12 h post-inoculation. Gene Ontologies were considered to be enriched when adjusted *p* value < 0.25 (Benjamini–Hochberg method) (Dataset 3), File S5: Enriched Gene Ontology terms that were categorised based on their occurrence within predefined ancestor/parent Gene Ontology terms related to immune system classes, as defined by CateGOrizer (Hu et al. 2008) (Dataset 4), File S6–Enriched Kyoto Encyclopedia of Genes and Genomes (KEGG) pathways based on lists of differentially expressed genes (*p* < 0.05 and fold-change ≥ 2) using zebrafish orthologs of rainbow trout transcripts. KEGG pathway over-representation analysis was performed using enrichKEGG (Benjamini–Hochberg correction, FDR < 0.1) and the KEGG terms of all zebrafish orthologs in the dataset as background (universe) (Dataset 5).
