# Supplementary material for: Evidence of Transcriptional Shutoff by Pathogenic Viral Haemorrhagic Septicaemia Virus in Rainbow Trout
Source: Viruses. 2021 Jun 11;13(6):1129. doi: 10.3390/v13061129 (PMC8231187; doi:10.3390/v13061129)

Evidence of transcriptional shutoff by pathogenic viral haemorrhagic septicaemia virus in rainbow trout

Irene Cano et al.

Supplemental Material (Tables and Figures)

Contents

Table S1. The number of Illumina read sequences obtained before and after quality-trimming.

Table S2. Summary of the rainbow (*Oncorhynchus mykiss)* genes and the nucleotide sequences of the primers and probes used for the Taqman qPCR assays.

Figure S1. Comparison of relative gene expression (fold-change) over time (3, 6 and 12 hours post inoculation) of rainbow trout cells (RTG-2) inoculated either with a pathogenic (J167) or a non-pathogenic (1p49) VHSV isolate relative to control cells by Taqman qPCR analysis or Illumina HiSeq sequence analysis.

Table S1. The number of Illumina read sequences obtained before and after quality-trimming.

Sample name	Isolate	hpi	Treatment ID	Replicate ID	Number of raw paired reads	Number of trimmed paired reads	Percentage of reads retained
CEFASR-0022-control_0_1	Control	0	Control	1p49_0_1	9,028,972	9,007,168	99.8%
CEFASR-0022-control_0_2	Control	0	Control	1p49_0_2	9,240,202	9,219,625	99.8%
CEFASR-0022-control_0_3	Control	0	Control	1p49_0_3	9,794,127	9,771,121	99.8%
CEFASR-0022-1p49_3_1	Non-pathogenic VHSV (1p49)	3	1p49_3	1p49_3_1	7,053,150	7,036,131	99.8%
CEFASR-0022-1p49_3_2	Non-pathogenic VHSV (1p49)	3	1p49_3	1p49_3_2	8,785,409	8,765,254	99.8%
CEFASR-0022-1p49_3_3	Non-pathogenic VHSV (1p49)	3	1p49_3	1p49_3_3	9,413,301	9,363,411	99.5%
CEFASR-0022-1p49_6_1	Non-pathogenic VHSV (1p49)	6	1p49_6	1p49_6_1	7,208,349	7,184,677	99.7%
CEFASR-0022-1p49_6_2	Non-pathogenic VHSV (1p49)	6	1p49_6	1p49_6_2	8,836,676	8,816,782	99.8%
CEFASR-0022-1p49_6_3	Non-pathogenic VHSV (1p49)	6	1p49_6	1p49_6_3	10,142,464	10,117,697	99.8%
CEFASR-0022-1p49_12_1	Non-pathogenic VHSV (1p49)	12	1p49_12	1p49_12_1	9,345,943	9,325,192	99.8%
CEFASR-0022-1p49_12_2	Non-pathogenic VHSV (1p49)	12	1p49_12	1p49_12_2	8,415,353	8,397,070	99.8%
CEFASR-0022-1p49_12_3	Non-pathogenic VHSV (1p49)	12	1p49_12	1p49_12_3	10,344,724	10,320,731	99.8%
CEFASR-0022-J167_3_1	Pathogenic VHSV (J167)	3	J167_3	J167_3_1	8,782,662	8,765,137	99.8%
CEFASR-0022-J167_3_2	Pathogenic VHSV (J167)	3	J167_3	J167_3_2	9,998,149	9,955,306	99.6%
CEFASR-0022-J167_3_3	Pathogenic VHSV (J167)	3	J167_3	J167_3_3	8,498,150	8,443,376	99.4%
CEFASR-0022-J167_6_1	Pathogenic VHSV (J167)	6	J167_6	J167_6_1	10,640,588	10,489,167	98.6%
CEFASR-0022-J167_6_2	Pathogenic VHSV (J167)	6	J167_6	J167_6_2	10,856,233	10,717,483	98.7%
CEFASR-0022-J167_6_3	Pathogenic VHSV (J167)	6	J167_6	J167_6_3	8,356,069	8,330,423	99.7%
CEFASR-0022-J167_12_1	Pathogenic VHSV (J167)	12	J167_12	J167_12_1	7,845,417	7,825,015	99.7%
CEFASR-0022-J167_12_2	Pathogenic VHSV (J167)	12	J167_12	J167_12_2	8,388,979	8,372,225	99.8%
CEFASR-0022-J167_12_3	Pathogenic VHSV (J167)	12	J167_12	J167_12_3	8,178,876	8,160,335	99.8%

Table S2. Summary of the rainbow (*Oncorhynchus mykiss)* genes and the nucleotide sequences of the primers and probes used for the Taqman qPCR assays. *Beta-actin* was used as reference gene for normalization purposes. PCR efficiency was calculated using the formula: Efficiency = $-1+10^{(-1/slope)}$.

Genbank Accession	Gene name	Gene symbol	Forward primer / Reverse primer	Probe Fam 5' - MGB 3'	PCR efficiency	Notes
AJ438158.1	*Beta-actin*	*actb*	GAAATCGCCGCACTGGTT / CGGCGAATCCGGCTTT	TTGACAACGGATCCGGT	103%	Reference gene
XM_021610144.1	*Adiponectin receptor protein 1-like*	*adipor1*	CCCTCCAGAGCTTCCCAAA / CCCGGACCCTTTCTATCACA	CAAGCTGTAGGAGGCTT	103%	Cell membrane receptor
XM_021586448.1	*Serum amyloid A-5 protein-like*	*saa*	GTACCGCTTCCCTGGTGAAG / CCGTATGCACGCCACATGT	TGCTCGAGGTGCTAGA	89%	Acute phase response
XM_021602847.1	*Heat shock protein 30-like*	*hsp30*	CACCTGCTCCCTGGCTCAT / ATGGATTCTTTGGTGCCTGAA	ACGGGAAGCTCCAC	98%	Cellular stress
XM_021597368.1	*Band 4.1-like protein 5*	*epb41l5*	CGGCCCGGTCCAGAAG / CACTGTAGCGGAAGCGTGATC	CAGCACGCTCTGGGT	93%	Cytoskeletal protein binding
NM_001124633.1	*CD209 molecule*	*cd209*	CCTTACCATCCTGCACAGCAA / CCTCCGGGCCTCTTTTTC	AAACAGCATGACGCTCT	96%	C-type lectin receptor/pathogen recognition

Figure S1. Comparison of relative gene expression (fold-change) over time (3, 6 and 12 hours post inoculation) of rainbow trout cells (RTG-2) inoculated either with a pathogenic (J167) or a non-pathogenic (1p49) VHSV isolate, relative to control cells, by Taqman qPCR analysis or Illumina HiSeq sequence analysis. Taqman qPCR data is expressed as average gene relative transcription of three replicates ±95% confident interval (CI). a) Adiponectin receptor protein 1-like (*adipor1*); b) Heat shock protein 30-like (*hsp30*); c) CD209 molecule (*cd209*); d) Serum amyloid A-5 protein-like (*saa*); and e) Band 4.1-like protein 5 (*epb41l5*). (*) represents significant differences (P ≤ 0.05) between groups at the same time post inoculation. Illumina sequencing data was extracted from Data Set 2 in the supplemental material.

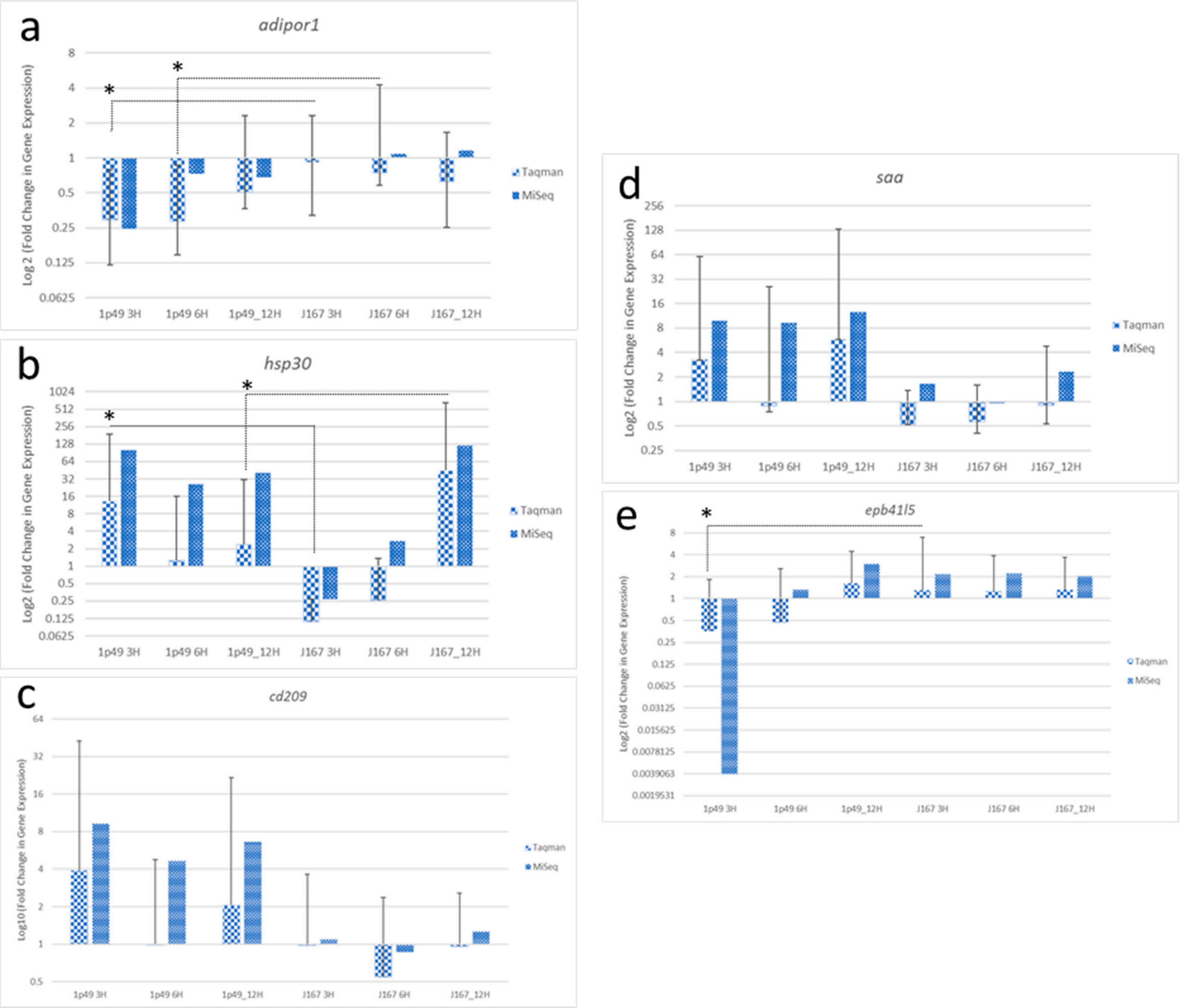

Supplement: Supplementary file 1 [file viruses-13-01129-s001.zip › Cano et al - Supplemental File 1 viruses.pdf]
